# Synergetic regulation of cancer cells and exhausted T cells to fight cold tumors with a fluorinated EGCG-based nanocomplex

**DOI:** 10.1186/s12951-023-02205-6

**Published:** 2023-11-14

**Authors:** Jinlin Zhang, Mingyue Wang, Doudou He, Liang Zhang, Tianqing Liu, Kaikai Wang

**Affiliations:** 1 Department of Pharmacy, Affiliated Cancer Hospital of Nantong University, Nantong University, Nantong, 226001 China; 2 School of Pharmacy, Nantong University, Nantong, 226001 China; 3 NICM Health Research Institute, Western Sydney University, Westmead, NSW 2145 Australia

**Keywords:** EGCG, Programmed death-ligand 1, Delivery system, Immunotherapy

## Abstract

Immune therapy that targets PD-L1 (programmed cell death-ligand 1) is attractive to augment immune response by breaking the programmed cell death-1 (PD-1)/PD-L1 axis. However, T cell exhaustion associated with insufficient T cells infiltration may diminish the efficacy of cancer therapy. Here, we report a novel delivery system of FEGCG/FPEI@siTOX composed of fluorinated EGCG (FEGCG) and fluorinated polyethyleneimine (FPEI) for delivery of small interfering RNA anti-TOX (thymus high mobility group box protein, TOX) to treat tumor and metastasis. In this way, the reduction in PD-L1 expression by FEGCG can promote T-cell function, while inhibition of TOX expression with siTOX can alleviate T-cell exhaustion. FPEI are designed to deliver siRNA with high efficiency and low toxicity compared to classical PEI. Integrating FEGCG, FPEI and siTOX into such a novel system resulted in excellent anti-tumor and antimetastatic effects. It is a promising delivery system and potential strategy for the treatment of “cold” tumors.

**Supplementary Information:**

The online version contains supplementary material available at 10.1186/s12951-023-02205-6.

## Introduction

Immune therapy represents a revolutionary landscape to treat cancers by activating the immune system of patients [1]. While the paradigm of immunotherapy (e.g. checkpoint blockade, adoptive cell therapy, and vaccines) is diversified, its ultimate goal is to ensure that there is adequate infiltration of T cells in tumors for cytotoxic death of cancer cells [2, 3]. For instance, it is possible to liberate and restore the instincts of T cells by targeting the axis of programmed cell death-1 (PD-1) /programmed cell death-ligand 1 (PD-L1) to disrupt the interaction of PD-1 expression at T cells surface and PD-L1 on cancer cells [4]. Nevertheless, also with PD-L1 antibody administration in the clinic, the immune response rate and the therapeutic efficacy remain limited in most malignancies. Exhaustion of T cells, which severely impairs the function and phenotypes of cytotoxic T cells when they are exposed to persistent infections and antigens, is one of the critical factors contributing to this condition [5–7]. Thus, developing a novel strategy that co-regulate cancer cells and exhausted T-cells to improve the immune reaction to treat cancer is still required.

Currently, the characterized molecular program of exhausted T cells has been explored to advance and deepen our understanding of the relationship between immune response and exhaustion [8]. One distinguishing feature is that the ability to release cytotoxic cytokines and molecules from cytotoxic T-cells, like interferon (IFN-γ), granzyme, tumor necrosis factor alpha (TNF-α) and perforin are impaired [9, 10]. Another difference is the increased inhibitory receptors on effector T cells, including LAG3 (Lymphocyte Activator 3), TIM3 (T-cell immunoglobulin and mucin domain containing protein 3) and CD160, in addition to common molecules of PD-1 and CTLA4 (Cytotoxic T Lymphocyte protein 4) [11, 12]. Moreover, the transcriptional regulatory network involves to regulate the production of exhausted T-cells, which includes TOX (thymus high mobility group box protein TOX), a crucial nuclear factor in the reprogramming of T-cells exhaustion [5], [13, 14]. In the deletion or absence of TOX, the station of exhausted T-cells cannot be maintained, and the secretion of cytotoxic molecules is elevated [15–17]. Therefore, the interference of TOX expression with small interfering RNA (siRNA) is appealing for preventing T-cell exhaustion.

The next critical issue in applying siTOX therapy to exhausted T cells is selecting an efficient and safe delivery system when taking into account the instability and inefficiency of siRNA in vivo [18]. Cationic polymers, which are commonly used as carriers, can form polyplexes with siRNA to protect it from degradation. Notably, the stability and transfection efficiency of polyplexes require polymers with high molecular weight, which may cause unexpected toxicity [19, 20]. Recently, it has been reported that EGCG ((-)-epigallocatechin gallate), a natural polyphenolic derived from green tea, can be used as a carrier to deliver siRNA via hydrogen-bond interactions with minimal toxicity [21]. Specifically, EGCG can act as a PD-L1 inhibitor to regulate the tumor cell responses to immunotherapy [22, 23]. Therefore, the combination of EGCG and siTOX represents a potential system to co-regulate cancer cells and exhausted T-cells [24, 25].

**Fig. 1:**
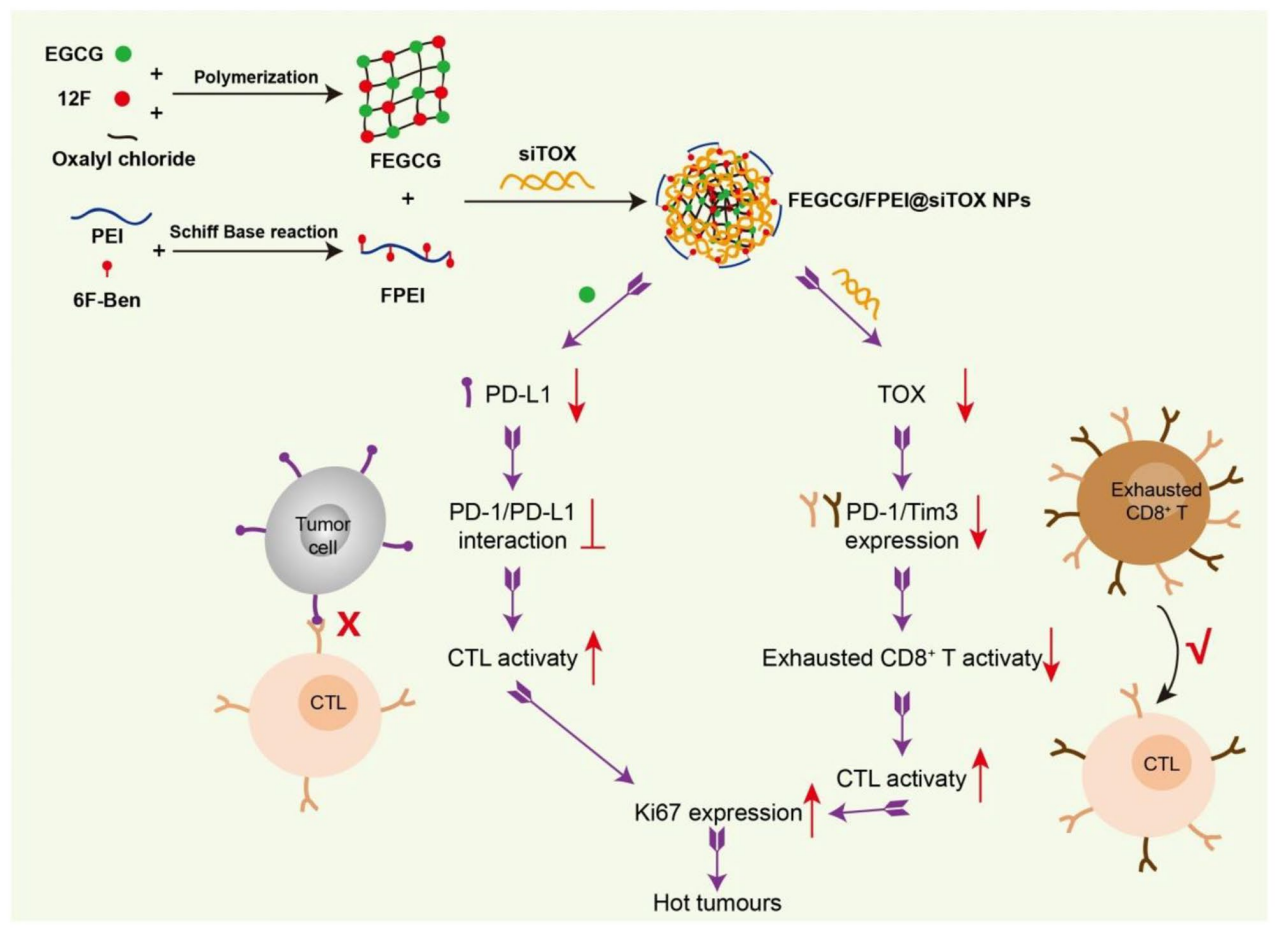
**Preparation and synergetic regulation mechanism of FEGCG/FPEI@siRNA NPs**. FEGCG and FPEI were synthesized and reacted to get FEGCG/FPI@siTOX NPs via electrostatic, hydrogen-bond, and fluorine interactions. The strategy to fight “cold” tumors depends on the synergetic regulation of tumor cells and exhausted T cells

Here, we first fluorinated and functionalized EGCG to produce the fluorinated EGCG (FEGCG) polymer, because the unique lipophobic and hydrophobic properties of fluorinated chains were beneficial for stability and transfection efficacy in siRNA delivery [26–28] (Fig. 1). Furthermore, fluorinated polyethyleneimine with low-molecular-weight (FPEI) was coated to form FEGCG/FPEI@siRNA nanoparticles (NPs) via electrostatic and fluorine interactions [29–31]. FEGCG/FPEI@siRNA NPs were prepared by electrostatic adsorption, hydrogen bonding and fluorine-fluorine interaction, resulting in good immunotherapeutic effect for the treatment of cancer. As a novel “green” NPs, FEGCG/FPEI@siRNA can not only decrease the expression of PD-L1to break the PD-1/PD-L1 interaction but also silence TOX expression to mitigate T cells exhaustion. The combined regulation of tumor cells and exhausted T-cells can boost T-cell infiltration and reverse “cold” tumors into “hot” tumors [32, 33]. With its efficacy demonstrated in the model of breast cancer, this novel system of FEGCG/FPEI@siRNA NPs will serve as a promising platform for the immunotherapy of cancer.

## Results and discussion

### Synthesis and Characterization of FEGCG, FPEI and FEGCG/FPEI@siRNA polyplexes

Fluoropolymers of FEGCG and FPEI were synthesized according to their synthetic route in Fig. S1-2. Their structure was demonstrated as shown in [1] H-NMR and [19] F-NMR (Fig. S3-4). The formation of FEGCG/FPEI@siRNA NPs should mainly depend on hydrogen-bond interactions, fluorine interactions and electrostatic interactions. The change in the fluorescence spectra of Cy5-siRNA was performed to demonstrate the involvement of hydrogen-bond interactions and fluorine interactions in the preparation of FEGCG/FPEI@siRNA NPs. As shown in Fig. S5A, Cy5-siRNA had a maximum fluorescence intensity at around 668 nm. After complexation with EGCG or PEI, the fluorescence intensity of Cy5-siRNA was decreased due to hydrogen-bond interactions or electrostatic interactions. After fluorination, the fluorescence intensity of Cy5-siRNA was further decreased compared with their parents. Meanwhile, the combination of FEGCG and FPEI achieved the best quenching effect on the fluorescence intensity of Cy5-siRNA. The siRNA release property of FEGCG/FPEI@siTOX NPs was further demonstrated, and results showed that FEGCG/FPEI@siTOX NPs exhibited the pH-dependent release of siRNA (cumulative release of 10% at pH 7.4, 20% at pH 6.8, and 39% at pH 5.0), which indicated that the NPs would be disassembled in endo/lysosomes after being taken up due to the cleavage of the Schiff base in FPEI (Fig. S5B).

**Fig. 2:**
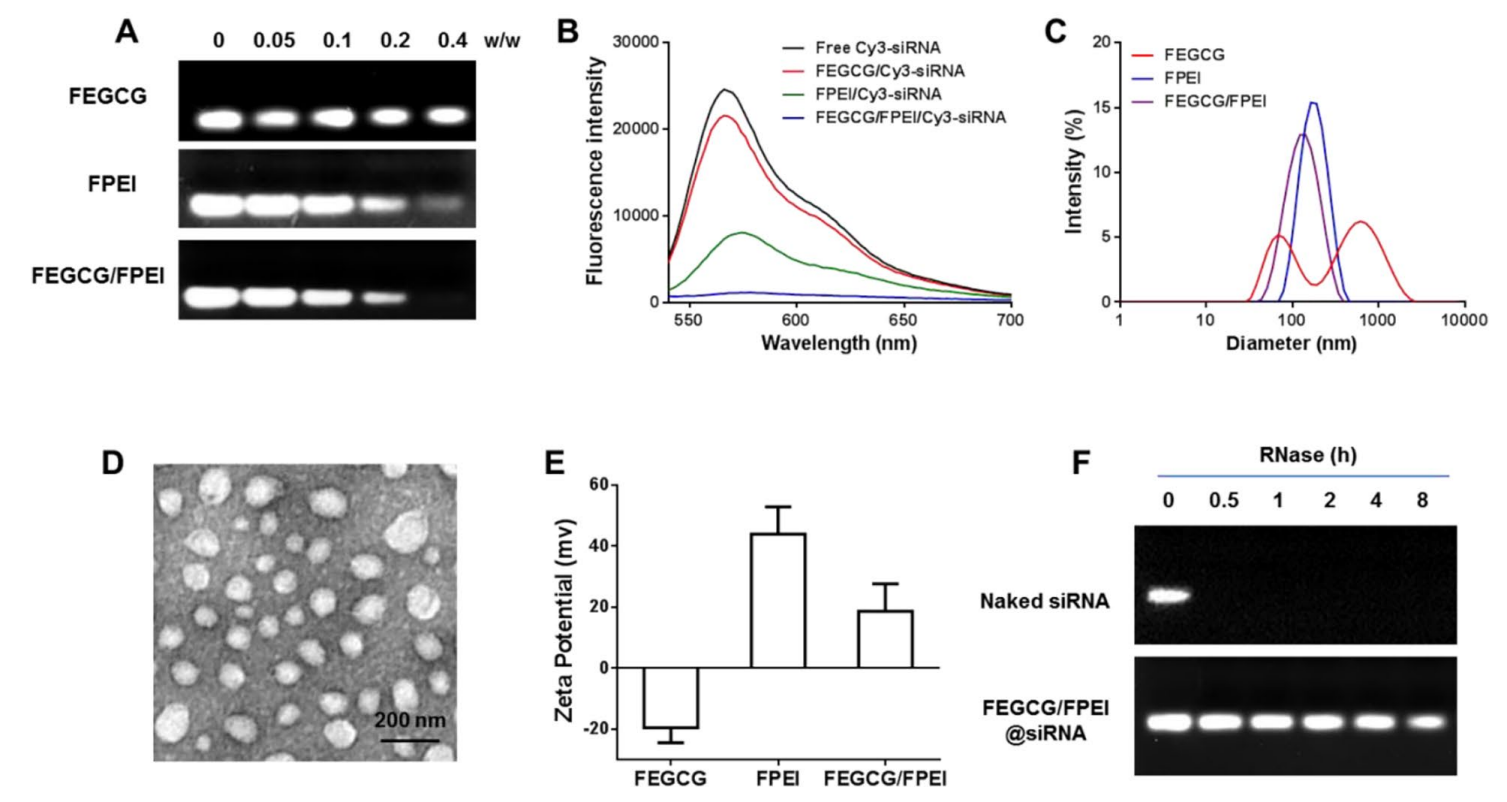
**Preparation and characterization of FEGCG/FPEI@siRNA NPs**. (**A**-**B**) siRNA condense ability of FEGCG, FPEI, and FEGCG/FPEI determined by agarose gel electrophoresis and fluorescence quenching assay. (**C**) Size distribution of FEGCG, FPEI and FEGCG/FPEI NPs. (**D**) Representative transmission electron microscope image of FEGCG/FPEI@siRNA NPs. (**E**) Zeta potential of FEGCG, FPEI and FEGCG/FPEI NPs. (**F**) RNase stability of FEGCG/FPEI@siRNA NPs at different time

Fluorescence quencher assay and agarose-gel electrophoresis were used to determine their siRNA condensation ability. As shown in Fig. 2A-B, FEGCG/FPEI completely condensed siRNA at w/w 0.4, while visual bonds remained in FEGCG and FPEI groups. Moreover, the fluorescence intensity of Cy3 was remarkably reduced when compared with FEGCG and FPEI groups at 570 nm. The particle size and distribution of FEGCG/FPEI@siRNA NPs were more uniform and smaller compared to those of FEGCG and FPEI groups, with an average size of 122 nm, 350 nm, and 164 nm, respectively (Fig. 2C). The transmission electron microscopic image showed the FEGCG/FPEI@siRNA NPs with spherical shape (Fig. 2D). Zeta potential of FEGCG/FPEI@siRNA nanoparticles with 18 mV was helpful for their safety compared with FPEI@siRNA NPs with 42 mV and crossing the cell membrane compared with FEGCG@siRNA NPs with − 20 mV (Fig. 2E). RNase stability revealed that FEGCG/FPEI prevented the siRNA degradation under RNase conditions (Fig. 2F). In addition, FEGCG/FPEI@siTOX NPs exhibited good stability against PBS (pH 7.4), 10% FBS or a combination of PBS (pH 7.4) and 10% FBS at 4℃ or room temperature (Fig. S5C). These findings suggest that combining FEGCG and FPEI can deliver siRNA with satisfactory binding affinity and stability.

### Cytotoxicity and cell uptake of FEGCG/FPEI@siRNA NPs

**Fig. 3:**
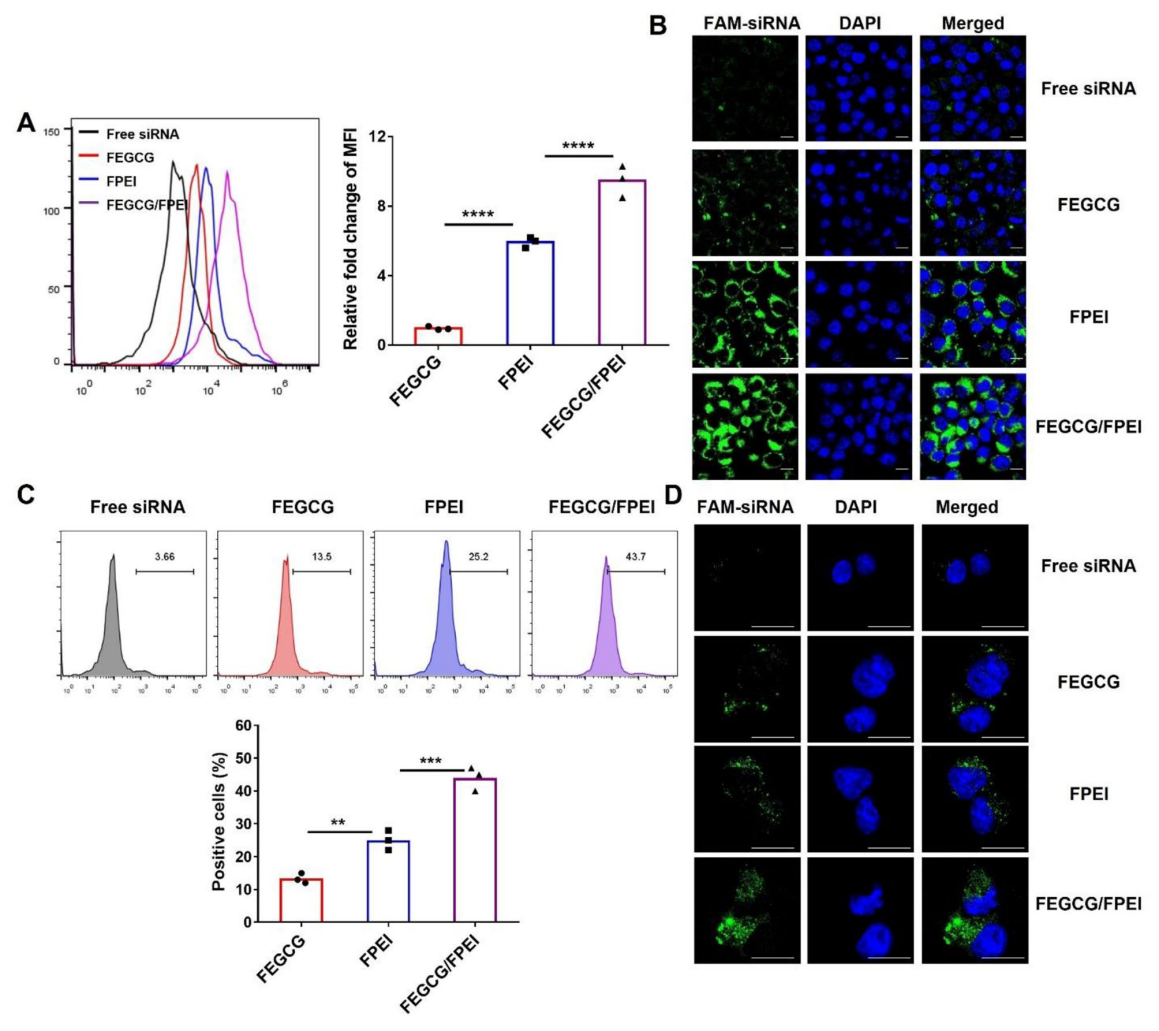
**Cellular uptake and intracellular trafficking of FEGCG/FFPI@siRNA NPs in 4T1 cells and exhausted T cells**. (**A**-**B**) Cellular uptake of FEGCG/FFPI@siRNA NPs detected by flow histogram and fluorescence intensity in 4T1 cells. (**C**-**D**) Flow cytometry assay and CLSM of the delivery efficacy in exhausted T cells after different treatments (scale bare = 10 μm). FEGCG, FPEI, and FEGCG/FPEI represented their corresponding siRNA NPs

Prior to using FEGCG/FPEI@siRNA NPs in siRNA delivery, the NPs cytotoxicity in 4T1 cells and exhausted T cells were firstly determined. As shown in Fig. S6A, the viability of 4T1 cells was significantly decreased after the fluorination on EGCG and PEI as compared to their parent controls. Meanwhile, the fluorination would increase the cytotoxicity of EGCG and PEI on T cells. Furthermore, the combination of FEGCG and FPEI induce an increase in cytotoxicity compared with the single treatment on two types of cells. As expected, fluorination enhanced the cellular uptake of siRNA in FEGCG and FPEI groups (Fig. S6B). Next, we demonstrated the superior delivery efficiency of siRNA with FEGCG/FPI as the carrier compared with FEGCG and FPEI. Flow cytometry and confocal microscopy were used to evaluate the cellular uptake of FEGCG, FPEI, and FEGCG/FPEI NPs in 4T1 cells. From the Fig. 3A-B, MFI (the mean fluorescence intensity) of FAM-siRNA in FEGCG/FFPI and FPEI groups was 9-fold and 6-fold higher than that in the FEGCG group, respectively. In addition, compared with FPEI NPs and FEGCG NPs groups, the green signals of FAM-siRNA were significantly increased in the FEGCG/FFPI NPs group. As shown in Fig. 3C-D that reflected on exhausted T cells, the positive cells increased from 13.5% of FEGCG treatment to 25.2% after the FPEI treatment and 43.7% after the FEGCG/FPEI treatment. CLSM results further confirmed the superior efficiency of FEGCG/FPEI combination on siRNA delivery. Furthermore, the Pearson’s correlation coefficient (PCC) was determined to evaluate the colocalization of green (FAM-siRNA) and red signals (lysosome). As shown in Fig. S6C, the PCC was 0.63, 0.56, 0.58, and 0.07 in groups of free siRNA, FEGCG@siRNA, FPEI@siRNA, and FEGCG/FPEI@siRNA, respectively, which indicated the efficient endosomal escape in FEGCG/FPEI@siRNA NPs. These findings indicate that combining FEGCG and FPEI is a promising strategy to deliver siRNA with acceptable safety and efficient delivery efficacy.

### Regulation of tumor cells and exhausted T-cells of FEGCG/FPEI@siTOX NPs in vitro

**Fig. 4:**
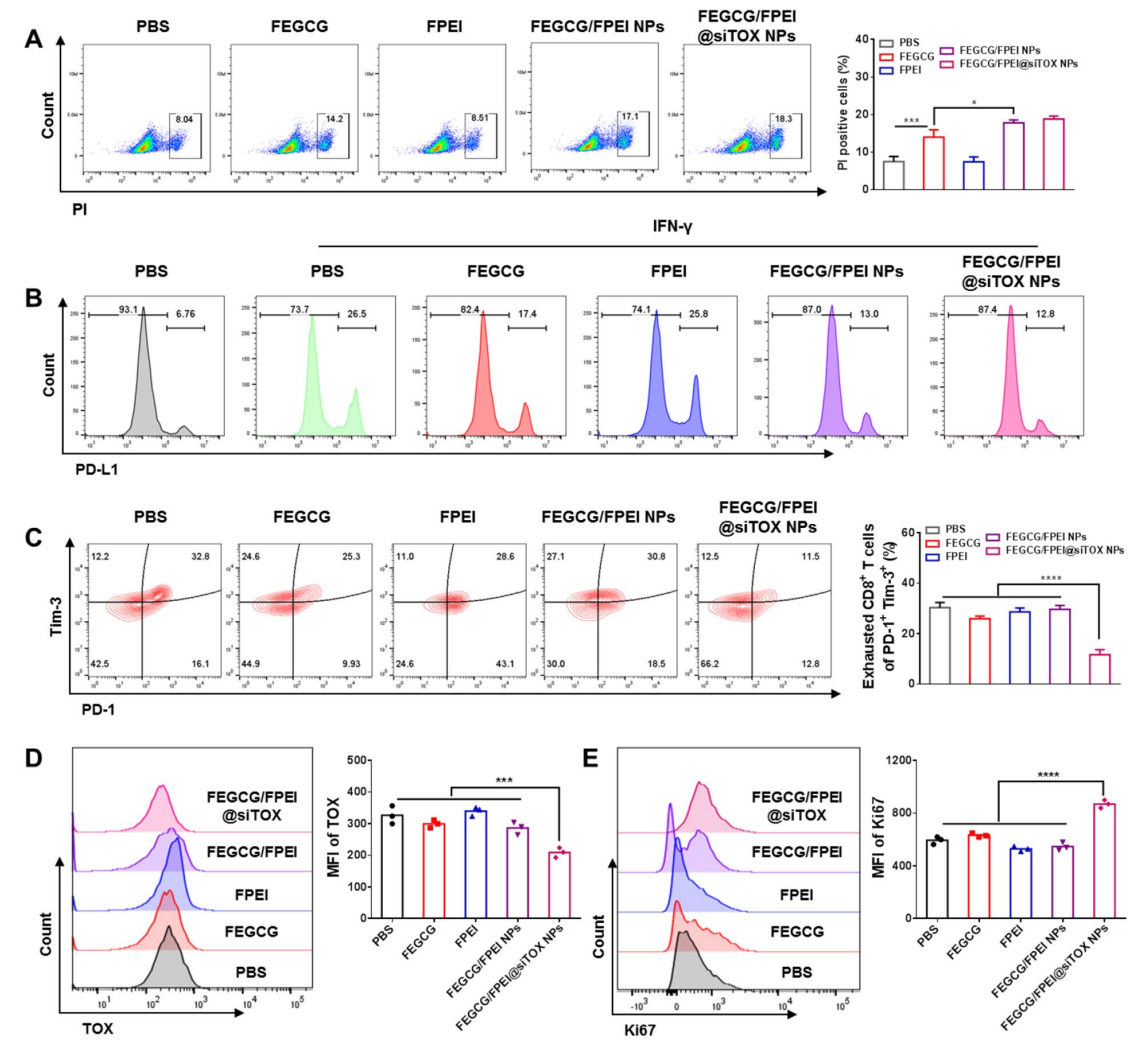
In vitro **co-regulation by decreasing PD-L1 expression and mitigating T cells exhaustion using FEGCG/FPEI@siTOX NPs**. (**A**) Apoptosis/necrosis ratio of different treatments in 4T1 cells. (**B**) PD-L1 expression of different treatments in 4T1 cells under IFN-γ stimulation in 4T1 cells. (**C**) Flow cytometry analysis of the percentage of PD-1^+^ Tim-3^+^ cells after different treatments in exhausted T cells. (**D**-**E**) TOX and Ki67 expression after different treatments in exhausted T cells

As designed, the co-regulation of tumor cells and exhausted T-cells by FEGCG/FPEI@siRNA NPs depends on the decreased PD-L1 expression by FEGCG and the silenced TOX expression by siTOX. Therefore, an apoptosis/necrosis ratio of 17.1% was induced by FEGCG/FPEI NPs, while PBS, FEGCG, and FPEI induced an apoptosis/necrosis ratio of 8.0%, 14.2% and 8.5% respectively in tumor cells. Interestingly, FEGCG/FPEI@siTOX NPs did not influence the apoptosis/necrosis ratio, in contrast to FEGCG/FPEI NPs (Fig. 4A). Subsequently, the expression of PD-L1 was examined, and the results showed that IFN-γ stimulation increased the expression of PD-L1 in the PBS group. FEGCG, FEGCG/FPEI NPs, and FEGCG/FPEI@siTOX NPs decreased PD-L1 expression from 26.5 to 17.4%, 13.0% and 12.8%, respectively. FPEI treatment, as anticipated, had no effect on PD-L1 expression compared with the PBS group (Fig. 4B). The effect of different treatments on PD-L1 expression was further confirmed by Western blot (Fig. S7). It is well known that IFN-γ can stimulate PD-L1 expression in the tumor microenvironment. To explain the potential mechanisms of EGCG on PD-L1 inhibition, the IFN receptor signaling pathway was explored by determining the expression of p-Akt and p-STAT1. As shown in Figure, the main treatments especially for FEGCG and FEGCG/FPEI@siTOX inhibited p-Akt and p-STAT1 expression in 4T1 cells (Fig. S8). These results indicate that EGCG regulated PD-L1 expression via inhibition of the Akt/STAT1 signaling pathway.

To demonstrate the siTOX treatment effects on the proportion and state of depleted T-cells, inhibitor molecules of Tim-3 and PD-1were selected to characterize the proportion of depleted T-cells. FEGCG/FPEI@siTOX NPs decreased PD-1^+^ Tim-3^+^ cells percentage from 32.8% in the PBS group to 11.5%. No significant effect on PD-1^+^ Tim-3^+^ cell percentage with other treatments not involving siTOX (Fig. 4C). This downregulation was attributed to the silenced TOX expression with siTOX from 100% of the PBS group to 63% of FEGCG/FPEI@siTOX NPs group as quantitated by flow cytometry. The effect of FEGCG/FPEI@siTOX NPs treatment on TOX expression was further confirmed by Western blot (Fig. S7). Finally, the state of T cell proliferation was examined using Ki67 as a biomarker, and FEGCG/FPEI@siTOX NPs treatment was found to increase Ki67 expression when compared to the PBS group (Fig. 4D-E) [34]. The above results showed that FEGCG/FPEI@siTOX NPs co-regulated PD-L1 expression and mitigated exhausted T cells.

### Biodistribution, antitumor and antimetastatic efficacy in vivo

**Fig. 5:**
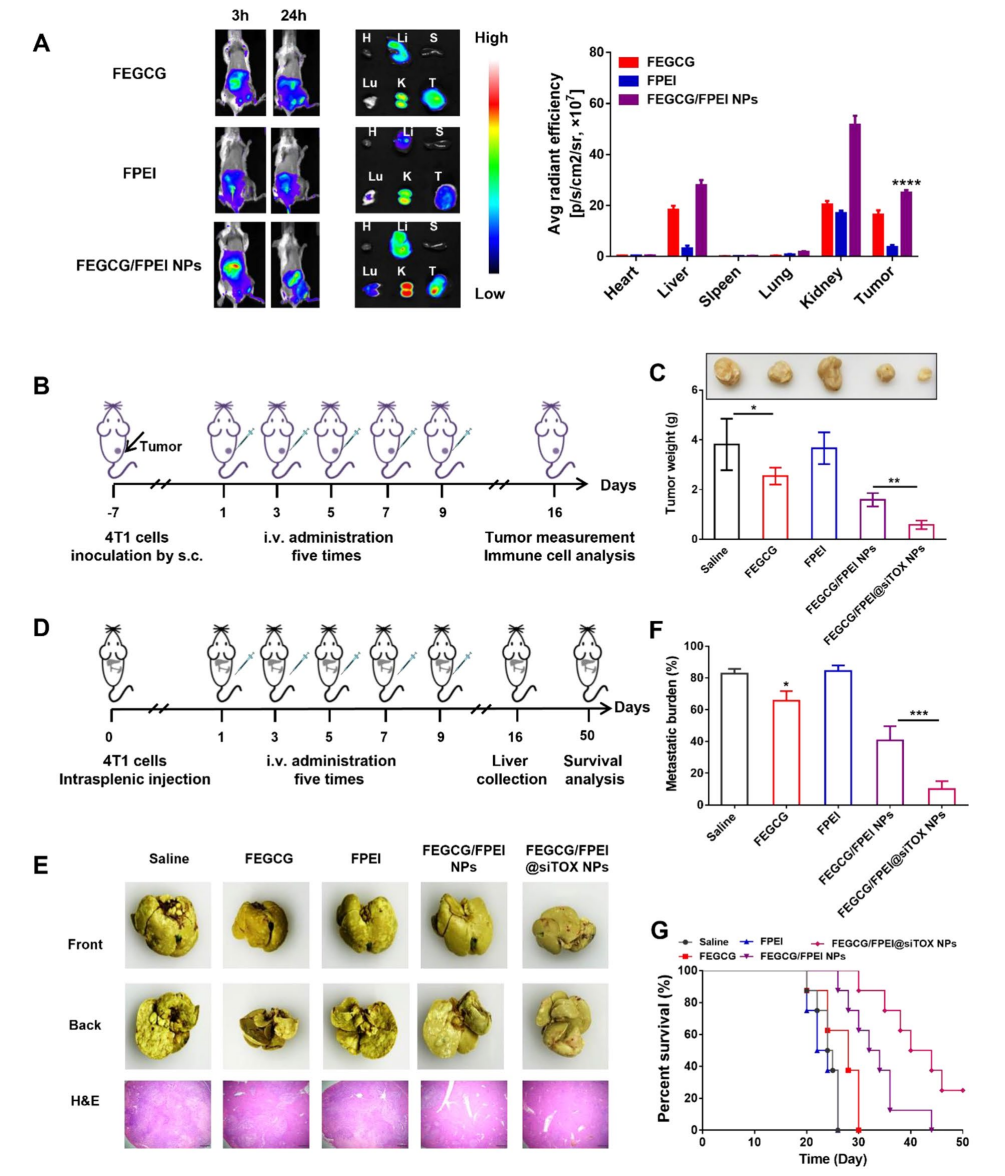
**Biodistribution, antitumor, and antimetastatic efficacy in vivo**. (**A**) Biodistribution of FEGCG@Cy5-siRNA, FPEI@Cy5-siRNA, and FEGCG/FPEI@Cy5-siRNA 3, and 24 h post-administration. Ex vivo images of major organs and quantitative fluorescence intensity (n = 3). Heart (H), liver (Li), spleen (S), kidney (K), lung (Lu), and tumor (T). (**B**-**C**) Treatment schedule and tumor weight after different treatments with saline, FEGCG, FPEI, FEGCG/FPEI NPs, and FEGCG/FPEI@siTOX NPs in subcutaneous tumor model (n = 3). (**D**-**E**) Treatment schedule, representative photographs, and H&E staining of liver tissues after different treatments with saline, FEGCG, FPEI, FEGCG/FPEI NPs, and FEGCG/FPEI@siTOX NPs in liver metastasis model (n = 3). (**F**-**G**) Quantification of metastatic burden and percent survival rates after different treatments in liver metastasis model (n = 8)

To assess the therapeutic efficacy of FEGCG/FPEI@siTOX NPs, a subcutaneous 4T1 tumor model was first established. The biodistribution of FEGCG/FPEI@Cy5-siRNA NPs was next investigated, and the results showed that FEGCG/FPEI NPs efficiently delivered Cy5-siRNA to the liver and tumor, as evidenced by stronger fluorescence intensity at these sites. Ex vivo images of major organs further supported the above results (Fig. 5A). Given that high accumulation of FEGCG/FPEI nanoparticles in the liver and kidney, their biosafety was further examined. H&E staining demonstrated that the main treatments didn’t induce changes in morphology compared with the saline group. Alanine aminotransferase (ALT) and creatinine (CR) as indicators of liver and kidney injury were selected to further examine the liver and kidney functions. As depicted in Fig. S9, the main treatments did not cause the change of ALT and CR levels compared with the saline group. These results indicated the acceptable biosafety of FEGCG/FPEI@siTOX NPs on liver and kidney, and the combination of FEGCG and FPEI had potential as a promising delivery system to deliver siRNA for in vivo cancer treatment.

The anti-tumor activity was then evaluated in the aforementioned cancer model, as the treat regimen was shown in Fig. 5B. The tumor cells were inoculated into the mice and the model was established when the volume of the tumor had reached to 200 mm [3]. All mice were sacrificed on day 16, and different NPs were administered on days 1, 3, 5, 7, and 9 for total of five times. The tumor growth and weight were significantly inhibited in treatment groups of contained FEGCG NPs, especially FEGCG/FPEI@siTOX NPs (Fig. 5C).

An intrinsically immunosuppressive microenvironment in the liver contributes to metastasis. [35, 36]. Therefore, we further evaluated FEGCG/FPEI@siTOX nanoparticles by a model of liver metastasis. The administration schedule was identical to that of the antitumor study, and the surviving experiment was under observation up to the 50th day (Fig. 5D). The burden of metastasis was significantly reduced, while the median survival time was prolonged after FEGCG/FPEI@siTOX NPs treatment. H&E staining images further demonstrated the superior antimetastatic efficacy of FEGCG/FPEI@siTOX NPs (Fig. 5E-G). These results support the antitumor and antimetastatic effects of the combination of FEGCG, FPEI, and siTOX-based delivery system.

### Immune activation in vivo

**Fig. 6:**
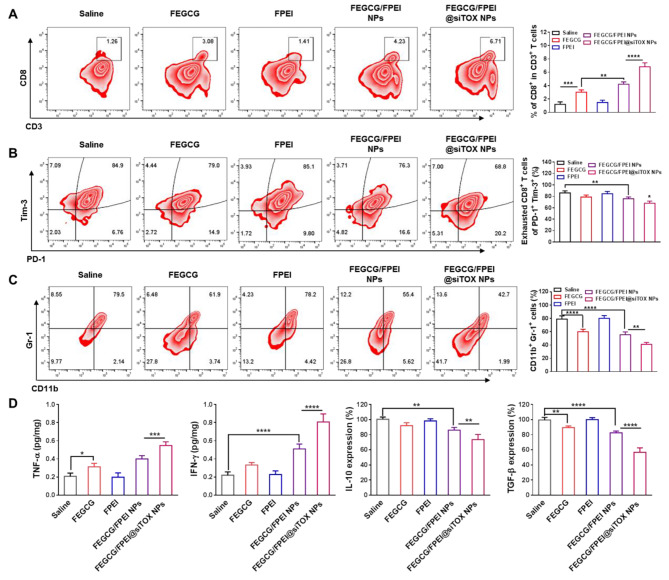
**Immune activation*in vivo***. (**A**) Percentage of CD45^+^ CD3^+^ CD8^+^ cells in lymphocytes after different treatments. (**B**) Percentage of PD-1^+^ Tim-3^+^ cells in T cells after different treatments. (**C**) Percentage of CD11b^+^ Gr-1^+^ cells in liver after different treatments. (**D**) TNF-α, IFN-γ, IL-10, and TGF-β levels in plasma after different treatments

To reveal the antitumor mechanism, tumor tissues and blood samples were collected to demonstrate effective immune activation [37]. T cell infiltration, as a crucial determinant of “hot tumors”, was first investigated by determination of the ratio of CD8^+^ cells and the expression of PD-L1 [38]. PD-L1 and TOX expression downregulation are contributed to the toxicity and proliferation of T cells. Treatments of FEGCG, FEGCG/FPEI NPs, and FEGCG/FPEI@siTOX NPs significantly decreased PD-L1 expression compared with the saline and FPEI groups (Fig. S10A and Fig. S11). The T-cells percentage increased to 6.7% after FEGCG/FPEI@siTOX NPs treatment, which was significantly higher than FEGCG/FPEI NPs treatment (4.2%), FPEI treatment (1.4%), FEGCG treatment (3.1%), and the control group (1.3%) (Fig. 6A and Fig. S10B). By counting the percentage of PD-1^+^ Tim-3^+^ cells, the proportion of exhausted T-cells was analyzed. The status of the exhausted T-cells was determined by the assessment of the expression of TOX and Ki67 (gated on CD8^+^ cells). According to Fig. 6B, both of FEGCG/FPEI NPs and FEGCG/FPEI@siTOX NPs remarkably reduced the PD-1^+^ Tim-3^+^ cells percentage in comparison to other groups. The decreased TOX expression in FEGCG/FPEI@siTOX NPs and increased Ki67 expression in both of FEGCG/FPEI NPs and FEGCG/FPEI@siTOX NPs further confirmed the above findings (Fig. S10C-D and Fig. S11). Myeloid derived suppressing cells, which are one of the suppressive immune cells characterized by CD11b^+^ and Gr-1^+^, were also inhibited after treatments with FEGCG, FEGCG/FPEI NPs, and FEGCG/FPEI@siTOX nanoparticles (Fig. 6C). Finally, the tumor necrosis factor-α (TNF-α) and interferon (IFN-γ) cytokines, and IL-10 and TGF-β inhibitory molecules were tested. Findings indicated that FEGCG/FPEI@siTOX NPs induced a robust immune response after combined PD-L1 and TOX inhibition by increasing in the levels of TNF-α and IFN-γ and decreasing in the levels of IL-10 and TGF-β (Fig. 6D).

## Conclusion

In summary, this study successfully developed a fluorinated EGCG-based nanocomplex (FEGCG/FPEI@siTOX) to co-regulate the expression of PD-L1 on tumor cells and the expression of TOX on exhausted T-cells. The FEGCG/FPEI@siTOX NPs were constructed through hydrogen-bond interactions, fluorine interactions, and electrostatic interactions, and they had satisfactory stability and siRNA delivery efficiency. Then, we demonstrated that the release of FEGCG and siTOX disrupted the PD-1/PD-L1 axis and alleviated the state of T-cell exhaustion, resulting in enhanced T-cell infiltration and associated immune response. A combination of these two approaches can achieve superior antitumor and antimetastatic efficacy [39–41]. Therefore, such a delivery system represents a promising platform and strategy for turning “cold” tumors into “hot” tumors in cancer immunotherapy.

## Materials and methods

### Materials

EGCG and 1 h,1 h,8 h,8 h-Perfluorooctane-1,8-Diol (12 F) was bought from Shanghai Macklin Biochemical Technology Co., Ltd (Shanghai, China). 3,5-bis-trifluoromethyl-phenyl)-methanone, PEI (600 Da), and Oxalyl chloride were purchased from Shanghai Aladdin Reagent Company (Shanghai, China). ELISA kits of TNF-α, IFN-γ, IL-10, and TGF-β were purchased from MultiSciences (Lianke) Biotech Co. Ltd. Fluorescent-labeled siRNA (Cy3, FAM, and Cy5 groups) and siTOX were obtained from GenePharma Co., Ltd. (Shanghai, China).

### Synthesis, preparation and characterization of FEGCG, FPEI, FEGCG/FPEI NPs and FEGCG/FPEI@siRNA NPs

FEGCG was prepared in accordance with the previous work [22]. Briefly, solutions of EGCG (459 mg, 1.0 mM), oxalyl chloride (212 mg, 1.67 mM), and 12 F (242 mg, 0.67 mM) were gently mixed and reacted for 6–12 h. The products were purified by evaporating, washing, and dialyzing to obtain FEGCG. FPEI was synthesized via the Schiff base reaction according to the previous report [15]. Briefly, PEI (600 mg, 1mM) and 6 F-Ben (726.3 mg, 3 mM) were dissolved and reacted with trace acetic acid in methanol for 24 h. The produced FPEI was purified by evaporating and dialyzing against deionized water. Their structures were demonstrated with [1] H-NMR and [19] F-NMR (Bruker 600 MHz).

FEGCG/FPEI NPs were prepared by mixing FEGCG and FPEI for 10 min before use (the concentrations of FEGCG and FPEI are 25 and 1 µg/mL, respectively). FEGCG/FPEI@siRNA NPs were produced through mixing FEGCG and siRNA at required w/w ratios for 10 min, and further coated with FPEI at the same ration of FEGCG/FPEI NPs for another 10 min. The capability of FEGCG, FPEI, and FEGCG/FPEI to condense siRNA was evaluated by agarose gel electrophoresis experiment. Briefly, agarose was dissolved in 0.5 × Tris/borate/EDTA buffer with Gel red to obtain the clear solution. Then, it was placed in a tank filled with related NPs, and performed at 100 mV for 15 min to visualize the siRNA bands. The size and zeta potential of FEGCG@siRNA NPs, FPEI@siRNA NPs, and FEGCG/FPEI@siRNA NPs were evaluated with the dynamic light scattering. The RNase stability of FEGCG/FPEI@siRNA NPs was measured by agarose gel electrophoresis. The morphology of FEGCG/FPEI@siRNA NPs was determined using a transmission electron microscope. The fluorescence spectra of Cy5-siRNA was detected using a fluorescence scanning spectrum with a multimode reader (excitation at 560 nm, emission at 620–740 nm). The colloidal stability of FEGCG/FPEI@siRNA NPs was evaluated by monitoring the size change at 0.5, 2, 12, 24, and 48 h in PBS (pH 7.4), 10% FBS or a combination of PBS (pH 7.4) and 10% FBS at 4 °C or room temperature. The siRNA release property of FEGCG/FPEI@siRNA NPs was demonstrated by detecting the fluorescence intensity of Cy5-siRNA.

### Cytotoxicity, cellular uptake and distribution

The cytotoxicity of different carriers was evaluated by a standard assay. Briefly, 4T1 cells or exhausted T cells were incubated with EGCG, FEGCG, PEI, FPEI, FEGCG/FPEI NPs, and FEGCG/FPEI@siTOX NPs for 24 h (the concentration of EGCG and FEGCG = 25 µg/mL, the concentration of PEI and FPEI = 1 µg/mL).

For cellular uptake and distribution study, 4T1 cells or exhausted T cells were seeded in 12-well plates for flow cytometry analysis or confocal dishes for confocal microscopic imaging. The cells were incubated with free FAM-siRNA, FEGCG@FAM-siRNA, FPEI@FAM-siRNA, and FEGCG/FPEI@FAM-siRNA NPs for 4 h (the concentration of FEGCG = 25 µg/mL, the concentration of FPEI = 1 µg/mL, and the concentration of siRNA = 2.5 µg/mL), and then stained with DAPI or lysotracker for further imaging.

### Animals and tumor models

The tumor model of 4T1-bearing mice was defined by subcutaneously injecting cells into the flank of female Balb/c mice. The metastasis model of 4T1-liver mice was constructed by intrasplenically injecting the 4T1 cells (5×10^5^) into the spleen of female Balb/c mice. All mice underwent partial splenectomy surgery.

### Expression of PD-L1 and the state of the exhausted T-cells in vitro

The PD-L1 silencing ability was evaluated by incubating pretreated 4T1 cells with IFN-γ (5 ng/mL) and further treating them with FEGCG, FPEI, FEGCG/FPEI NPs, and FEGCG/FPEI@siTOX NPs for 24 h. The expression of PD-L1 was determined by Western blot or staining with an anti-PD-L1-APC antibody and followed by flow cytometry analysis. In addition, the expression of (phosphorylation)-Akt and (phosphorylation)-STAT1 on 4T1 cells detected by Western blot after different treatments.

Changes in the proportion of exhausted T cells was analyzed by determining the PD-1 and Tim-3 levels. The expression of TOX and Ki67 was also determined by flow cytometry and Western blot in exhausted T cells.

The established 4T1 tumor was excised and digested with collagenase II, and then exhausted T cells were isolated from tumor tissues through the gradient Percoll solution method, and purified through the CD8 Positive Selection Kit (STEMCELL, Vancouver, Canada). Then, T cells were incubated with the treatments of FEGCG, FPEI, FEGCG/FPEI NPs, and FEGCG/FPEI@siTOX NPs for 24 h, and then stained with anti-CD8, anti-Tim3, anti-PD-1, anti-Ki67, and anti-TOX antibodies to analyze the above biomarkers with flow cytometry.

### Biodistribution study

Cy5 labeled siRNA was applied as a fluorescent group to prepare FEGCG@Cy5-siRNA NPs, FPEI@Cy5-siRNA NPs and FEGCG/FPEI@Cy5-siRNA NPs (10 mg/kg FEGCG, 0.4 mg/kg FPEI, and 1 mg/kg siRNA). Established mice were randomly divided the above groups and receiving the i.v. NP injection for once. The fluorescence images were captured at 3 and 24 h after injection. All mice were sacrificed at 24 h after the imaging to excise major organs for quantifying the fluorescence intensity. The liver and kidney were collected for H&E staining, and the blood was collected for ALT and CR analysis.

### Antitumor study in vivo

The mice were inoculated with 4T1 cells on day − 7. On day 0, mice of ~ 200 mm [3] tumor volume were randomized into five groups of normal saline, FEGCG, FPEI, FEGCG/FPEI NPs, and FEGCG/FPEI@siTOX NPs (10 mg/kg FEGCG, 0.4 mg/kg FPEI, and 1 mg/kg siTOX). Different treatments were performed on the 1st, 3rd, 5th, 7th and 9th day, and the mice were killed on 16th day to conduct biochemical analysis. The tumor volume was monitored as:

$$\text{T}\text{u}\text{m}\text{o}\text{r} \text{v}\text{o}\text{l}\text{u}\text{m}\text{e} \left({\text{m}\text{m}}^{3}\right)=0.5\times \text{l}\text{e}\text{n}\text{g}\text{t}\text{h}\times {\text{w}\text{i}\text{d}\text{t}\text{h}}^{2}$$

Tumor tissues were digested to collected lymphocytes and exhausted T cells. The former was incubated with anti–CD3, anti–CD8, anti–CD4, and anti–CD45 to determine the proportion of infiltrated T cells. The later was stained with anti-CD8, anti-Tim3, anti-PD-1 to count the ratio of PD-1^+^/Tim3^+^ cells, and stained with anti-TOX and anti-Ki67 to quantify TOX and Ki67 expression. Levels of TNF-α, IFN-γ, IL-10, and TGF-β in blood samples were analyzed with related ELISA kits. The infiltrated lymphocytes were also stained with anti-CD45, anti-CD11b, and anti-Gr-1 antibodies to determine the proportion of myeloid-derived suppressor cells. PD-L1 expression and TOX expression were detected by Western blot.

The intrasplenic surgery was conducted to establish the liver metastasis model on day 0, and the schedule was also performed on days 1, 3, 5, 7, and 9. On day 16, all mice were sacrificed to excise the liver. The tissue was fixed in Bouin’s solution to further statistic on the tumor metastasis burden and perform H&E staining. For survival analysis, the remaining mice were followed until day 50.

### Statistical analysis

The statistical analysis is carried out using the two-tailed Student’s t-test for the two groups. And one-way analysis of variance (ANOVA) was for multiple groups. The data were considered to be statistically significant when P < 0.05.

### Electronic supplementary material

Below is the link to the electronic supplementary material.

Supplementary Material 1

## Data Availability

The data used and analyzed during this study are included in this article and are available from the first authors on reasonable request.
