# Supplementary material for: Synergetic regulation of cancer cells and exhausted T cells to fight cold tumors with a fluorinated EGCG-based nanocomplex
Source: J Nanobiotechnology. 2023 Nov 14;21:420. doi: 10.1186/s12951-023-02205-6 (PMC10644671; doi:10.1186/s12951-023-02205-6)
Supplement: Supplementary file 1 — Supplementary Material 1 [file 12951_2023_2205_MOESM1_ESM.docx]

**Synergetic regulation of cancer cells and exhausted T cells to fight cold tumors with a fluorinated EGCG-based nanocomplex**

Jinlin Zhang^1,2^, Mingyue Wang^1,2^, Doudou He^1,2^, Liang Zhang^1,2^, Tianqing Liu^3*^, Kaikai Wang^1,2*^


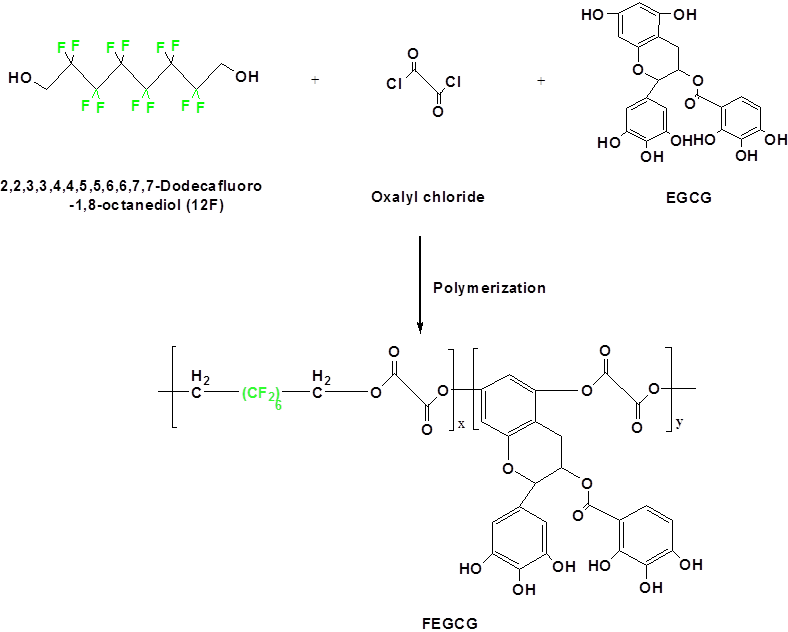


Figure S1. Synthetic route of FEGCG.


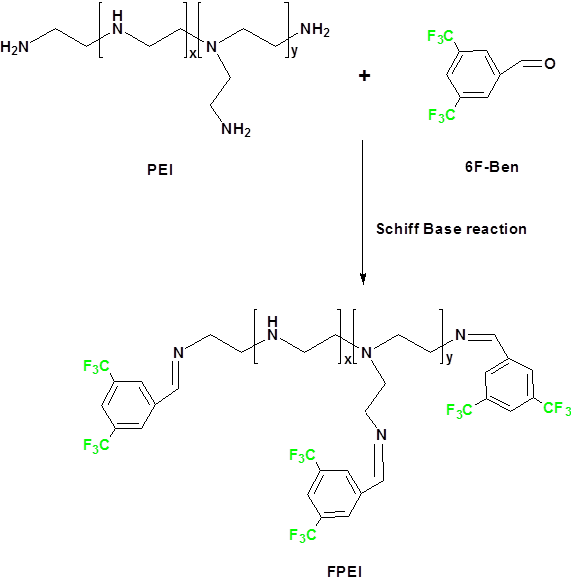


Figure S2. Synthetic route of FPEI.


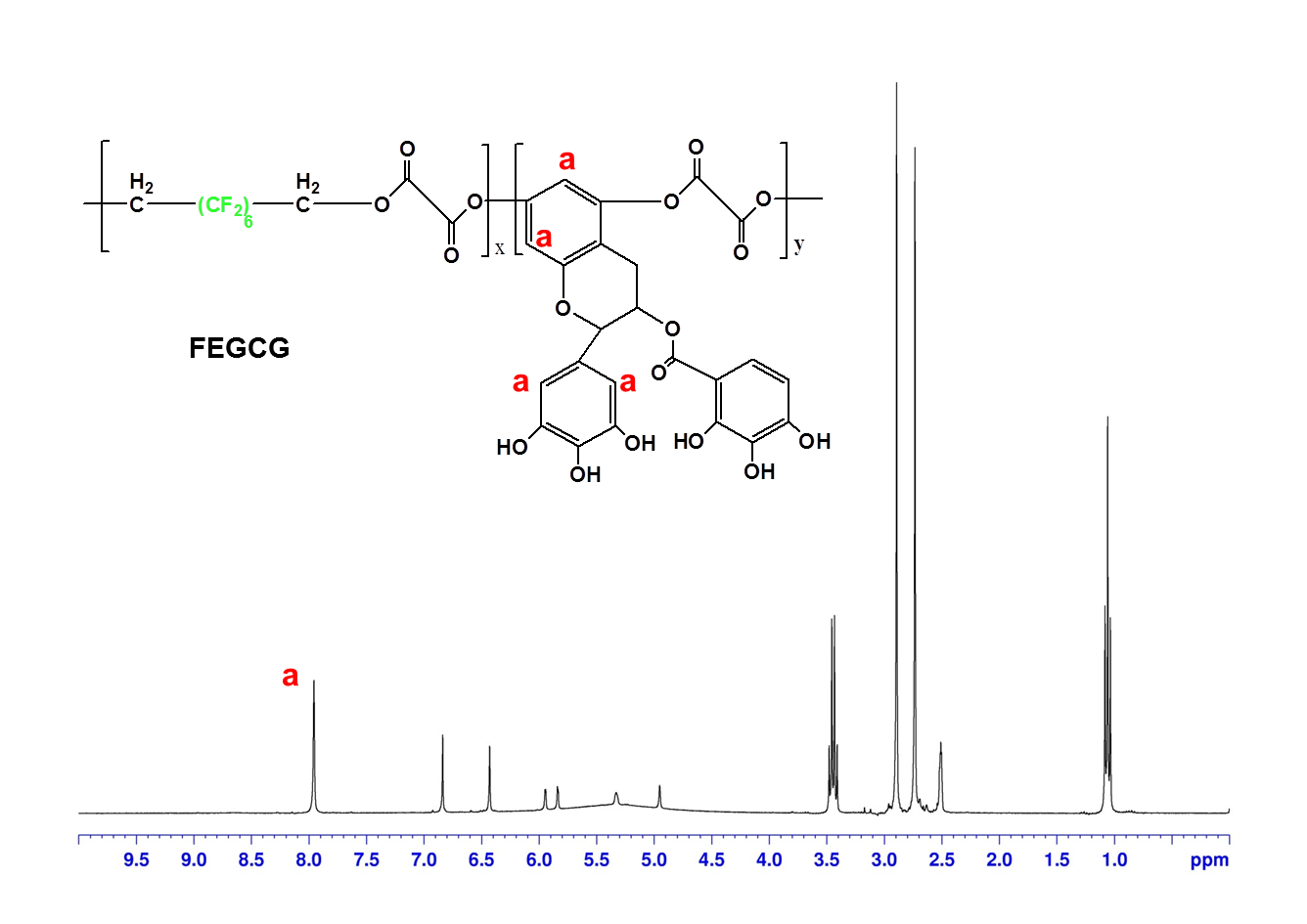


Figure S3. ^1^H-NMR of FEGCG in DMSO-d6.


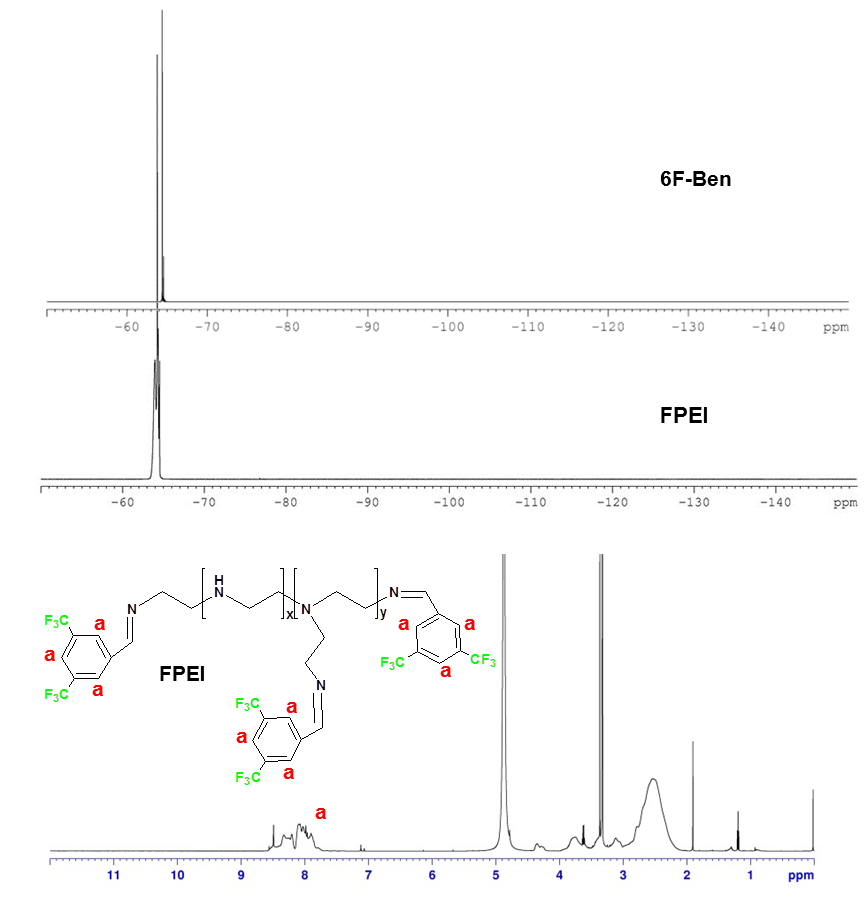


Figure S4. ^1^H-NMR and ^19^F-NMR of FPEI in D_2_O.


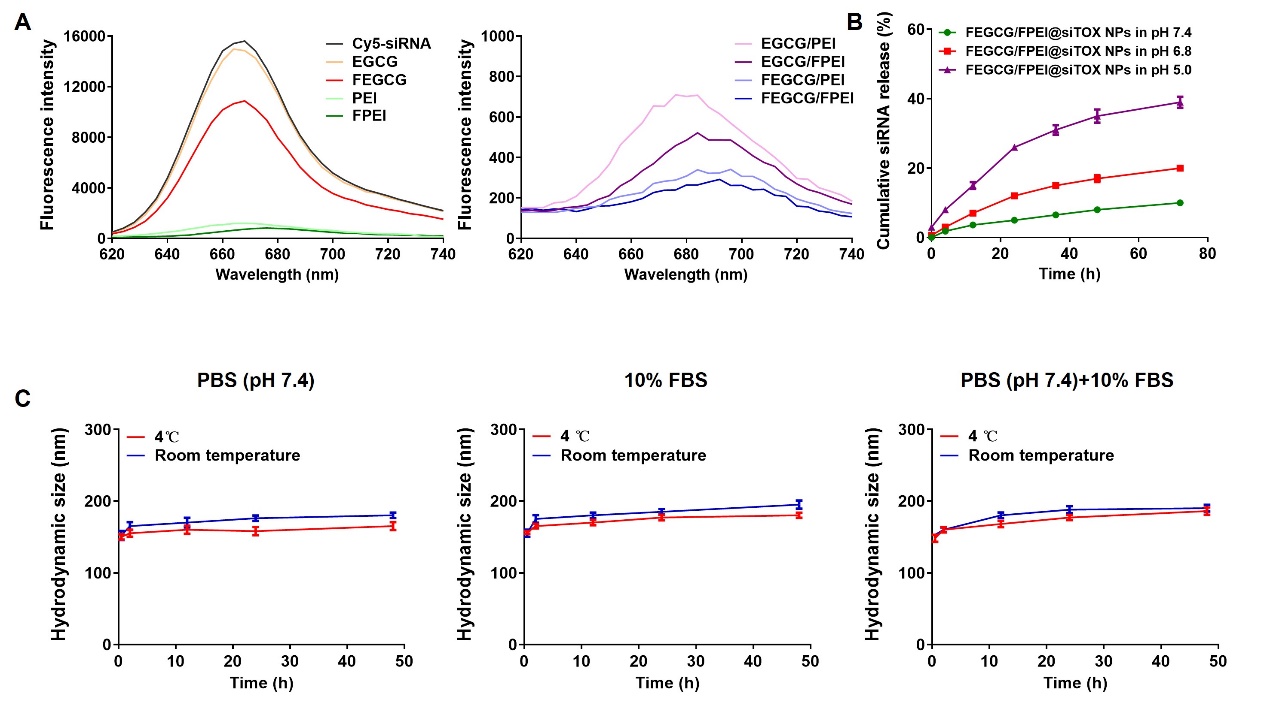


Figure S5. (A) Fluorescence spectra of Cy5-siRNA, EGCG@siRNA, FEGCG@siRNA, PEI@siRNA, FPEI@siRNA, EGCG/PEI@siRNA, EGCG/FPEI@siRNA, FEGCG/PEI@siRNA, and FEGCG/FPEI@siRNA. (B) siRNA release property of FEGCG/FPEI@siTOX NPs at different pH values. (C) Stability of FEGCG/FPEI@siTOX NPs against PBS (pH 7.4), 10% FBS or the combination of PBS (pH 7.4) and 10% FBS at 4°C or room temperature.


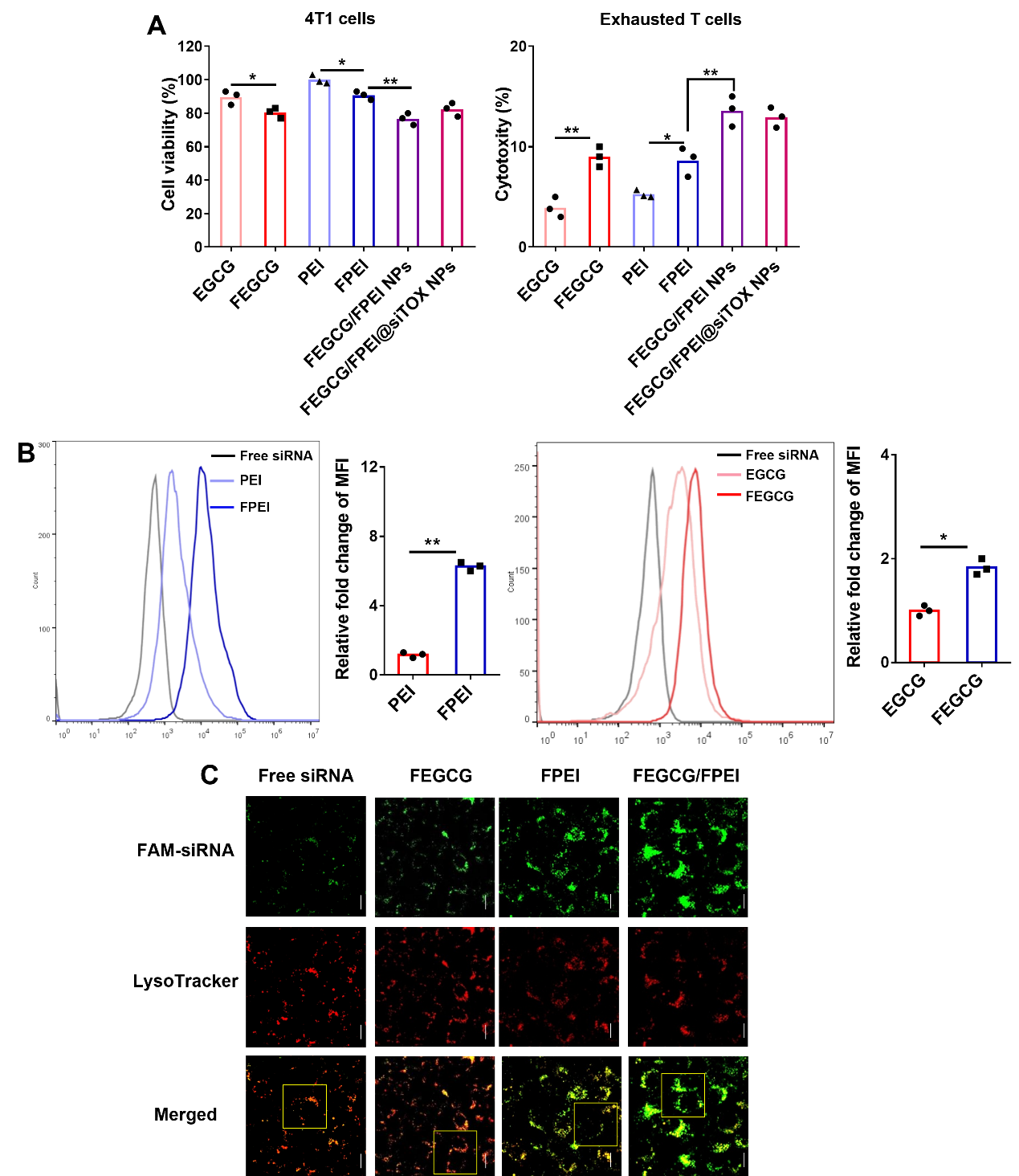


Figure S6. (A) The cell viability and cytotoxicity of EGCG, FEGCG, PEI, FPEI, FEGCG/FPEI NPs, and FEGCG/FPEI@siTOX NPs in 4T1 cells and exhausted T cells. (B) Cellular uptake of EGCG, FEGCG, PEI, and FPEI in 4T1 cells. (C) Intracellular distribution and trafficking of FEGCG/FFPI@siRNA NPs detected by CLSM in 4T1 cells. The Pearson’s correlation coefficient in indicated areas analyzed by ImageJ. FEGCG, FPEI, and FEGCG/FPEI represented their corresponding siRNA NPs.


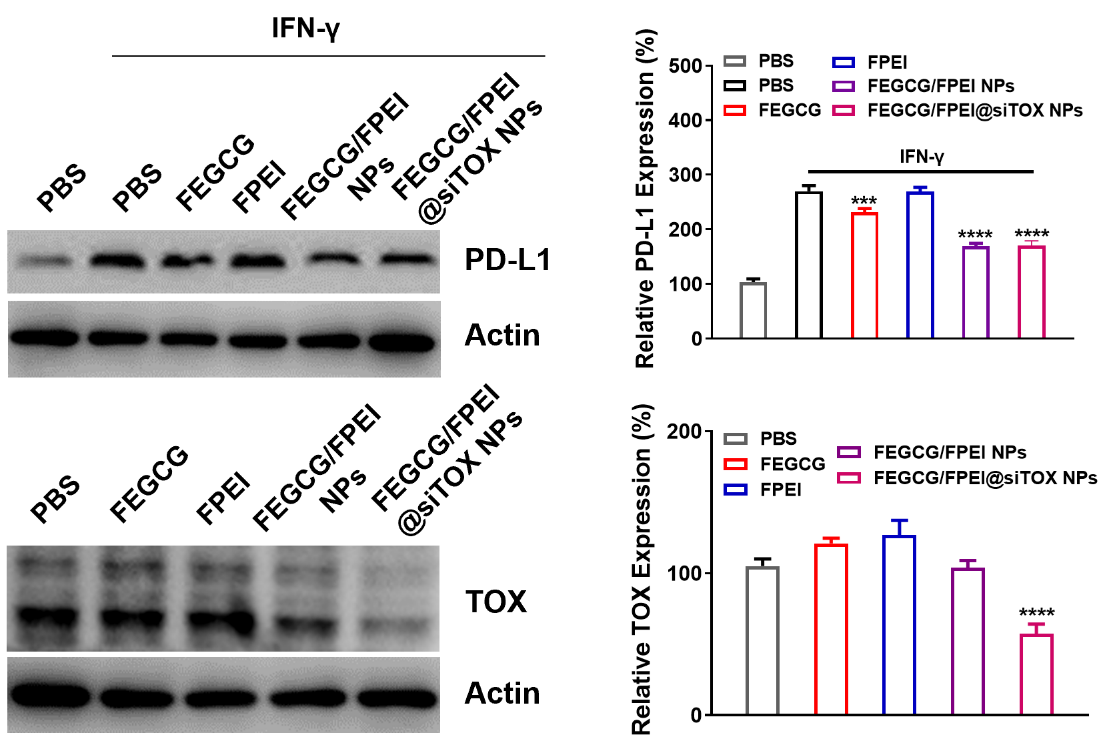


Figure S7. PD-L1 expression on 4T1 cells and TOX expression on exhausted T cells detected by Western blot after different treatments.


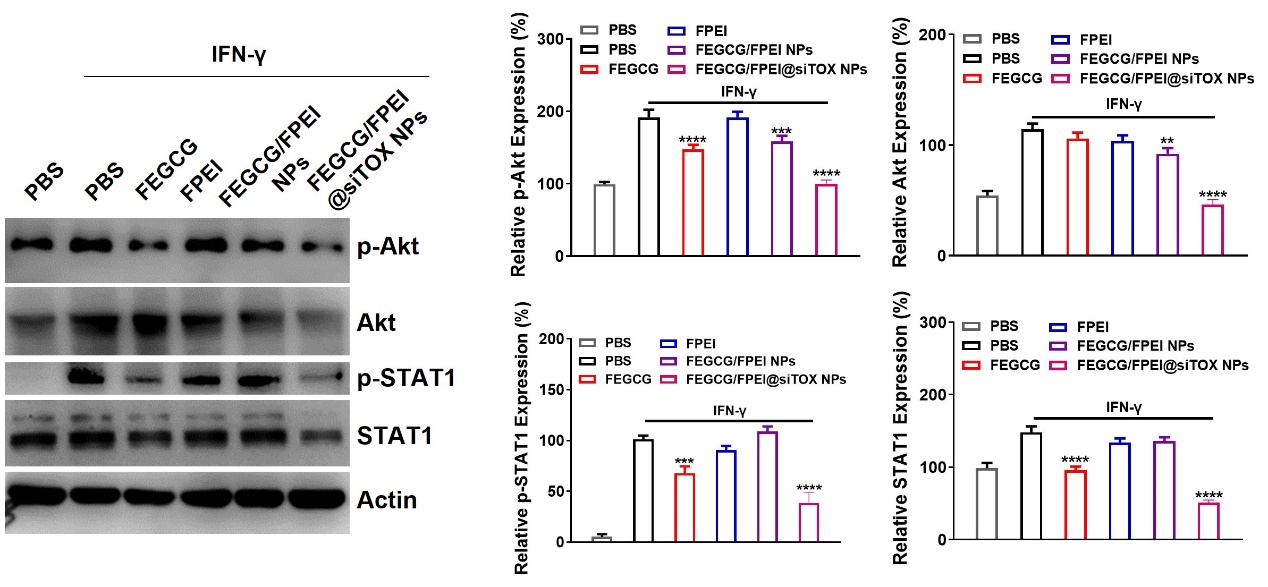


Figure S8. The expression of (phosphorylation)-Akt and (phosphorylation)-STAT1 on 4T1 cells detected by Western blot after different treatments.


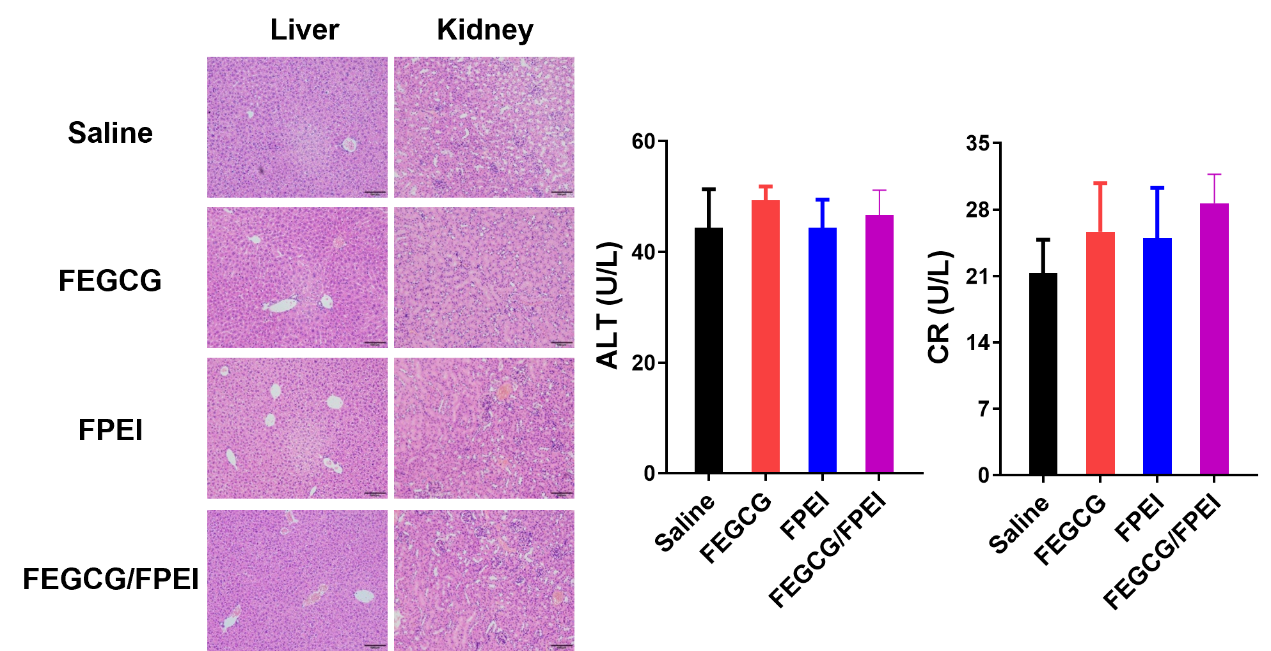


Figure S9. H&E staining of liver and kidney, and ALT and CR levels after different treatments in mice (scale bare=200 μm).


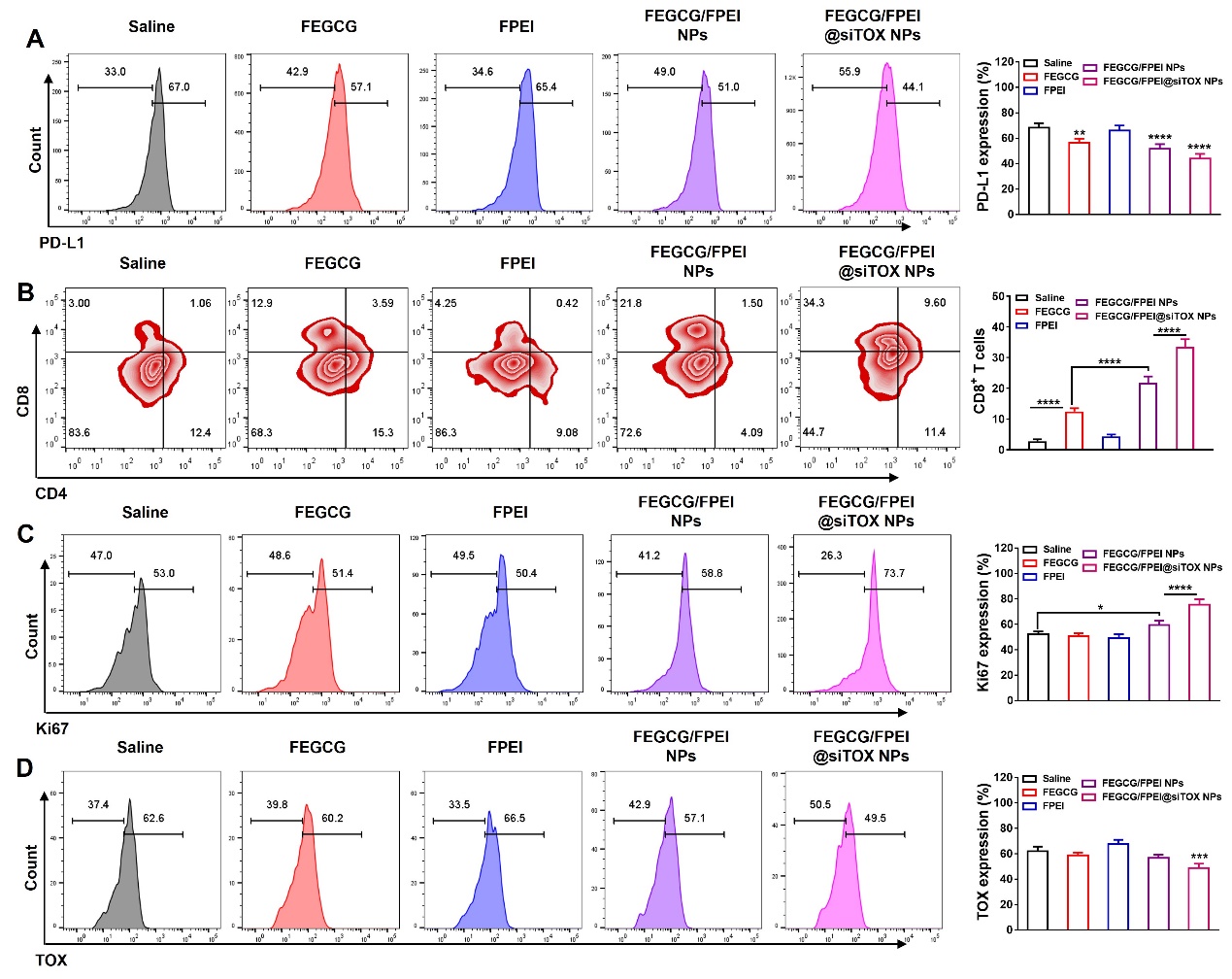


Figure S10. Expression of (A) PD-L1, (B) CD8, (C) Ki67, and (D) TOX after different treatments.


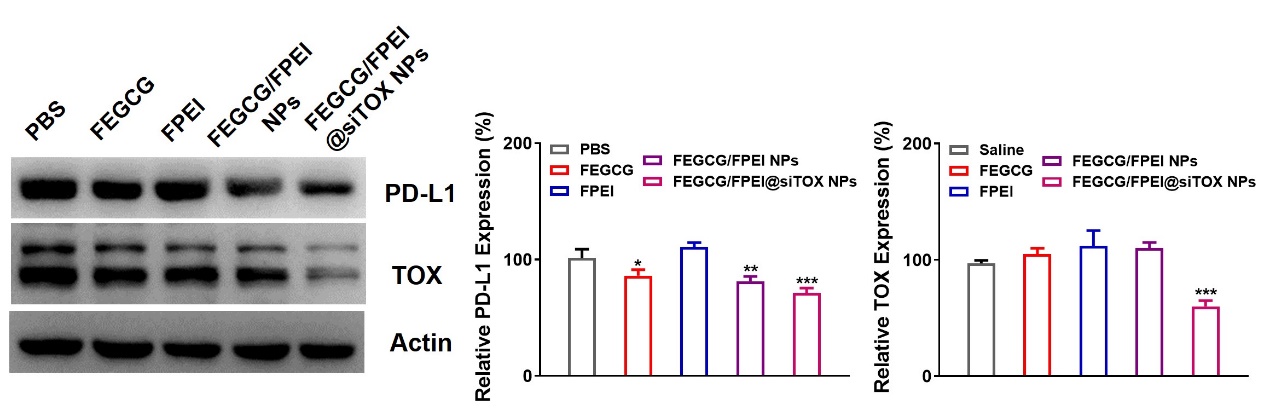


Figure S11. PD-L1 expression and TOX expression detected by Western blot after different treatments *in vivo*.
